# Supplementary material for: Feasibility of Photodynamic Therapy for Glioblastoma with the Mitochondria-Targeted Photosensitizer Tetramethylrhodamine Methyl Ester (TMRM)
Source: Biomedicines. 2021 Oct 13;9(10):1453. doi: 10.3390/biomedicines9101453 (PMC8533469; doi:10.3390/biomedicines9101453)
Supplement: Supplementary file 1 [file biomedicines-09-01453-s001.zip › biomedicines-1385115-supplementary/Supp Files.pdf]

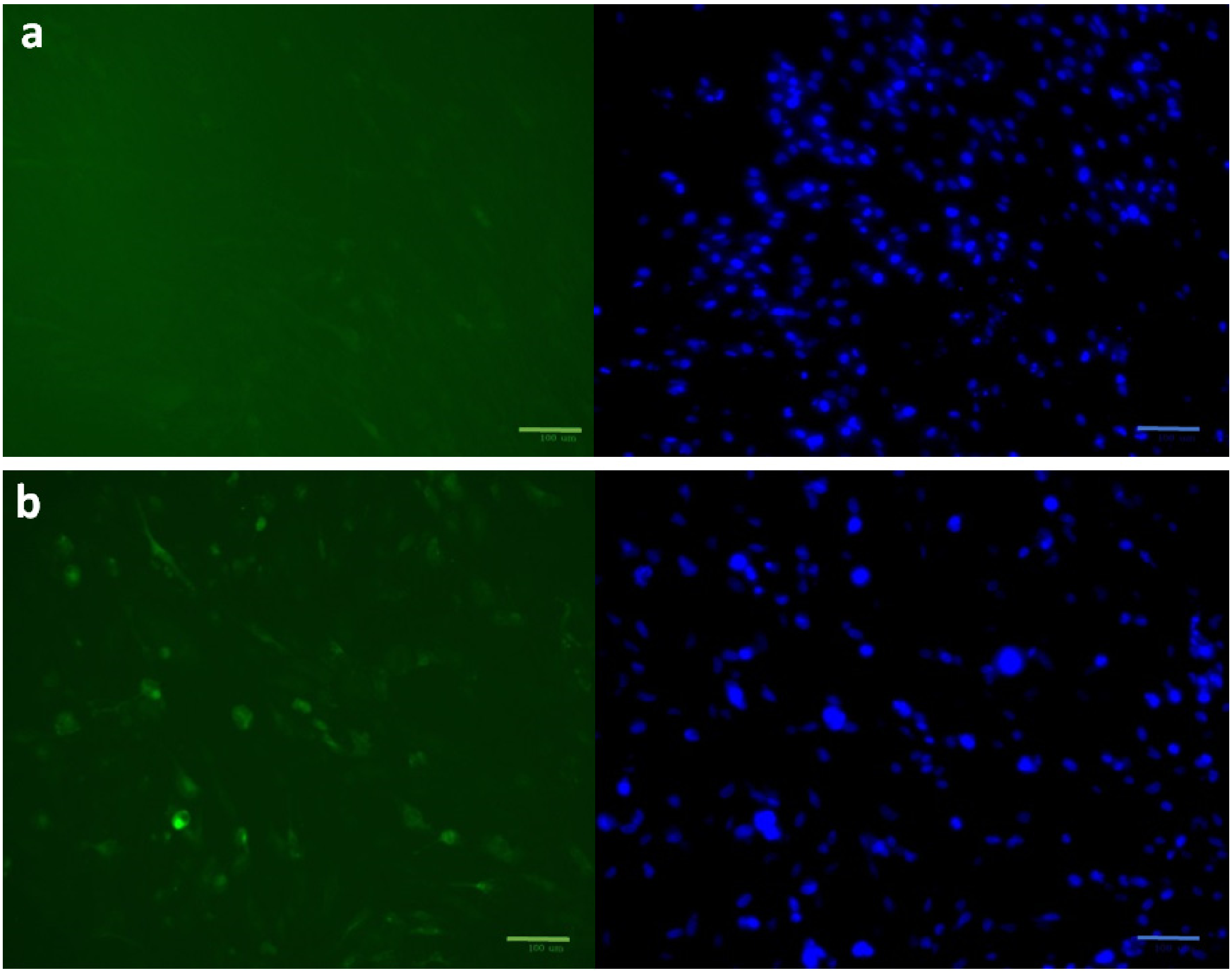

Figure S1. Activation of caspase reporter in surviving UP007 cells 10 days after TMRM-PDT. **(a)** TMRM-loaded control, no photoactivation **(b)** TMRM-PDT with photoactivation. Note that after TMRM-PDT the density of cells was greatly reduced and nuclei were enlarged, typical of apoptosis. Scale bar 100 µm.

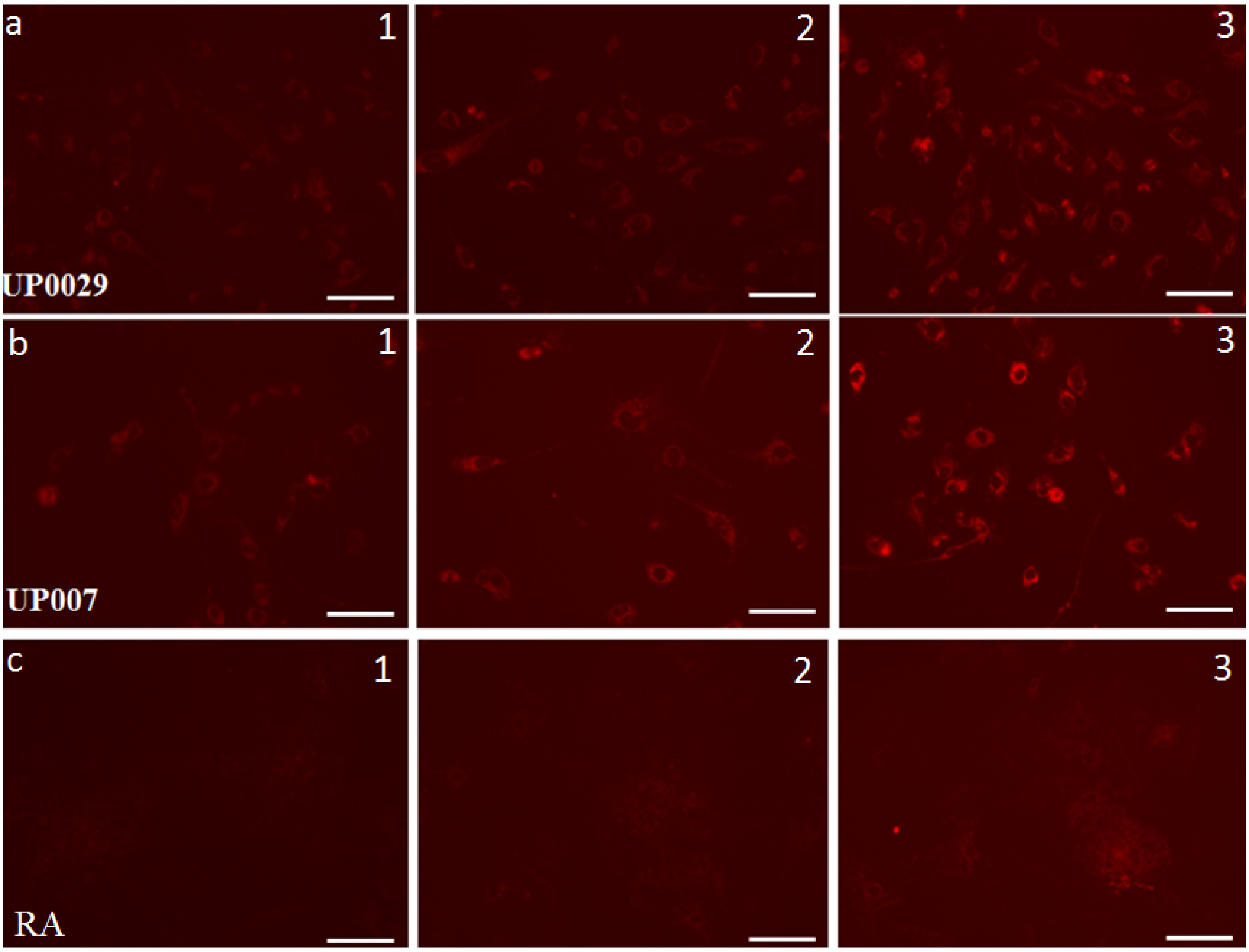

Figure S2. Images of GBM cells and rat astrocytes taken 24 hours after 40-minute loading with different concentration of TMRM. Fluorescence in GBM cells persists after 24 hours, but not in astrocytes. (a) UP007 GBM cells, (b) UP029 GBM cells, (c) rat astrocytes. (1) 100nM, (2) 300nM, (3) 800nM. Scale bar 100 nm

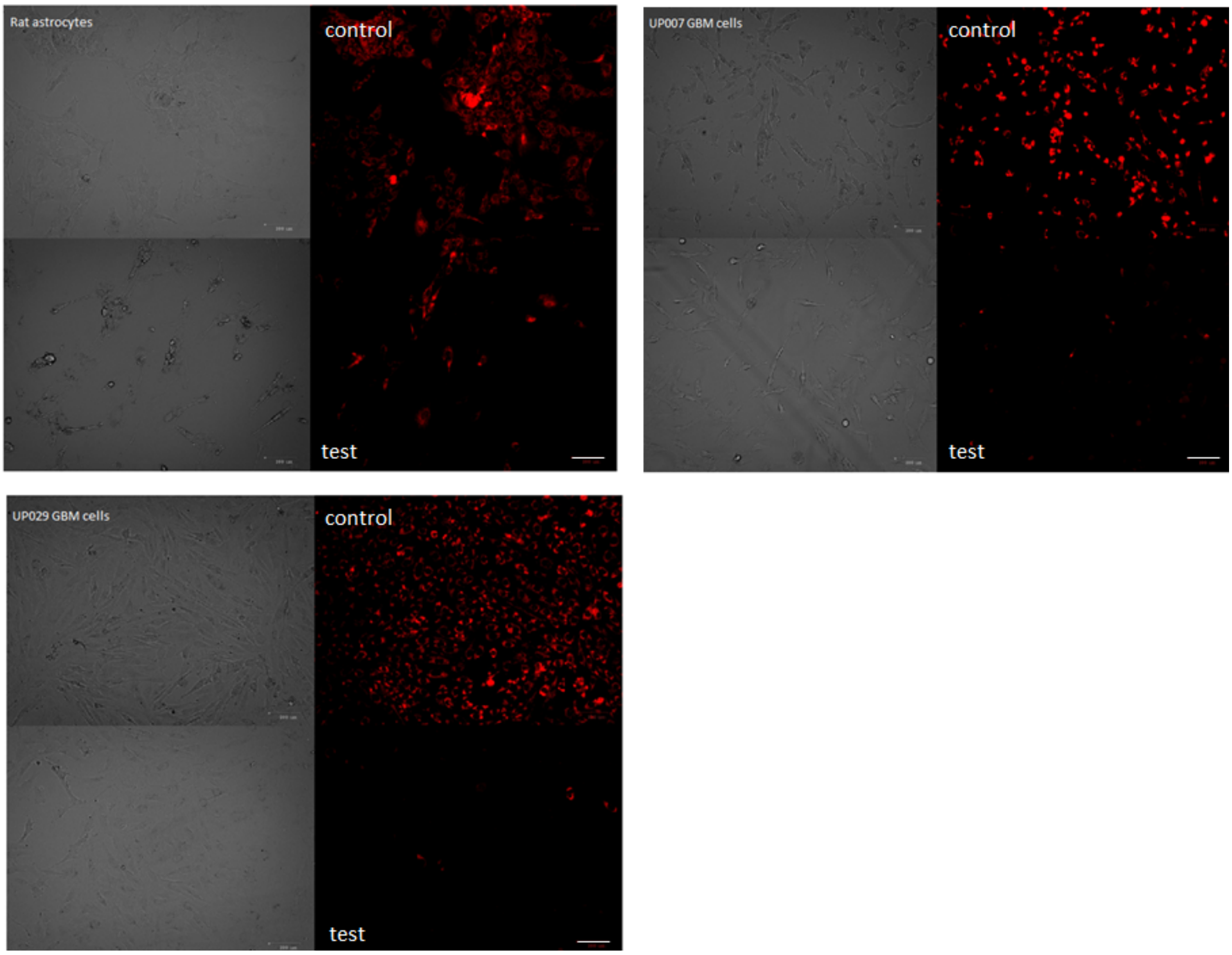

Figure S3. Loss of MMP in GBM cells persists 24 hours after photodynamic treatment. Comparing fluorescence intensity between normal rat astrocytes and GBM cells 24 hours after photodynamic injury. Grey scale images and corresponding fluorescent images are shown. With no photoactivation (control; upper panels), both cell types are brightly stained with TMRM. 24 hours after photoactivation (test; lower panels), rat astrocytes seem to have recovered their normal MMP as evident by stronger florescence signal than in GBM cells. Note that cell density of all cells is lower in test compared to controls. Scale bar= 100 µm

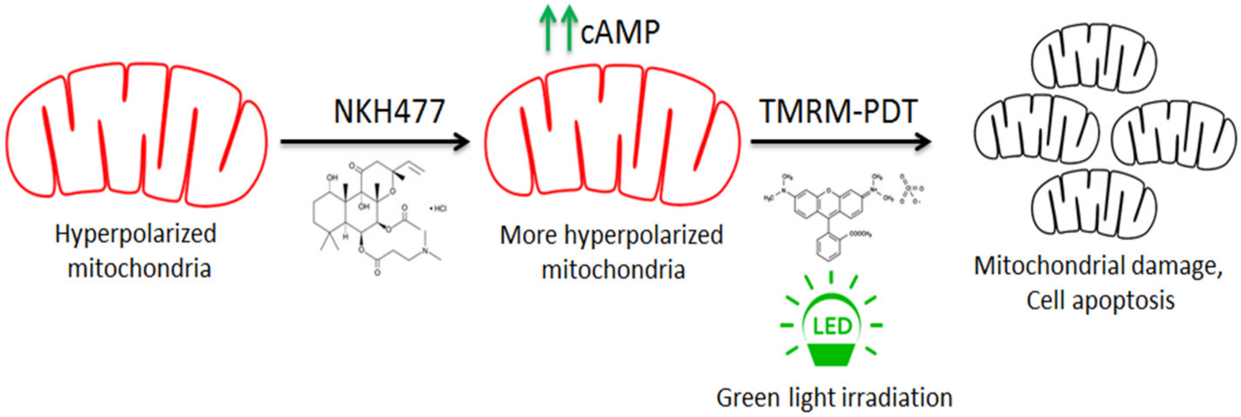

Figure S4: Hypothetical mechanism of action of the combined NKH477 and TMRM-PDT regimen.

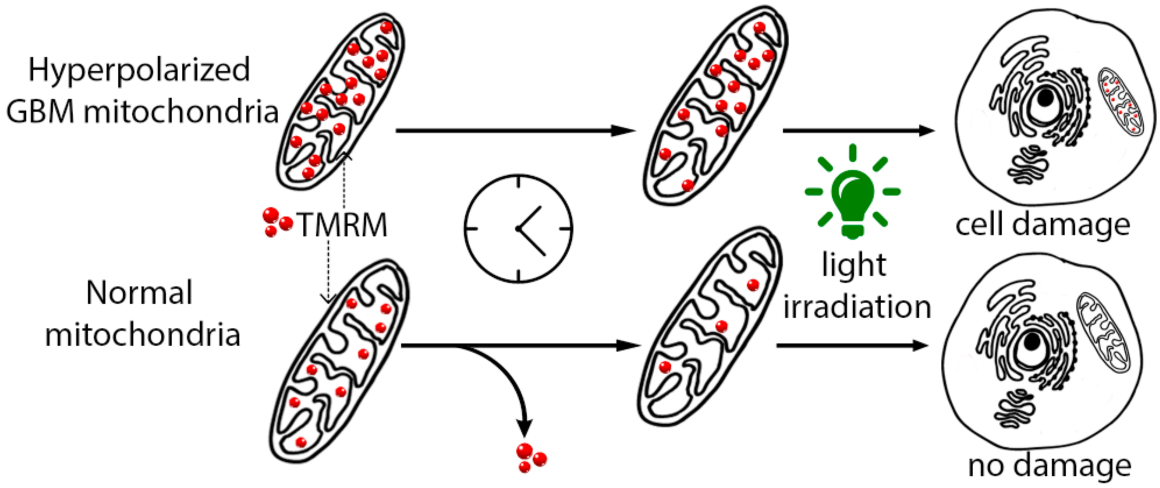

Figure S5: Schematic representation of the mechanism of delayed TMRM-PDT.
